# LiCl-promoted-dehydration of fructose-based carbohydrates into 5-hydroxymethylfurfural in isopropanol†

**DOI:** 10.1039/d0ra08737h

**Published:** 2021-01-05

**Authors:** Hao Ma, Zhenzhen Li, Lili Chen, Junjiang Teng

**Affiliations:** College of Chemistry, Guangdong University of Petrochemical Technology Maoming 525000 P. R. China thma@gdupt.edu.cn; School of Chemistry and Chemical Engineering, South China University of Technology Guangzhou 510640 P. R. China

## Abstract

The carbohydrate-derived 5-hydroxymethylfurfural (HMF) is one of the most versatile intermediate chemicals, and is promising to bridge the growing gap between the supply and demand of energy and chemicals. Developing a low-cost catalytic system will be helpful to the production of HMF in industry. Herein, the commercially available lithium chloride (LiCl) and isopropanol (i-PrOH) are used to construct a cost-effective and low-toxic system, *viz.*, LiCl/i-PrOH, for the preparation of HMF from fructose-based carbohydrates, achieving ∼80% of HMF yield under the optimum conditions. The excellent promotion effect of LiCl on fructose conversion in i-PrOH could be attributed to the synergistic effect of LiCl with i-PrOH through the LiCl-promoted and i-PrOH-aided dehydration process, and the co-operation of LiCl and i-PrOH for stabilizing the as-formed HMF by hydrogen/coordination bonds, giving a low activation energy of 68.68 kJ mol^−1^ with a pre-exponential factor value of 1.2 × 10^4^ min^−1^. The LiCl/i-PrOH system is a substrate-tolerant and scalable catalytic system, fructose (scaled up 10 times), sucrose, and inulin also give 73.6%, 30.3%, and 70.3% HMF yield, respectively. Moreover, this system could be reused 8 times without significant loss of activity. The readily available and low-toxic LiCl, the sustainable solvent (i-PrOH), the renewable starting materials, and the mild reaction conditions make this system promising and sustainable for the industrial production of HMF in future.

## Abstract 2

Fructose-based carbohydrates can be dehydrated into HMF in the LiCl-promoted and i-PrOH mediated system efficiently.

## Graphical abstract

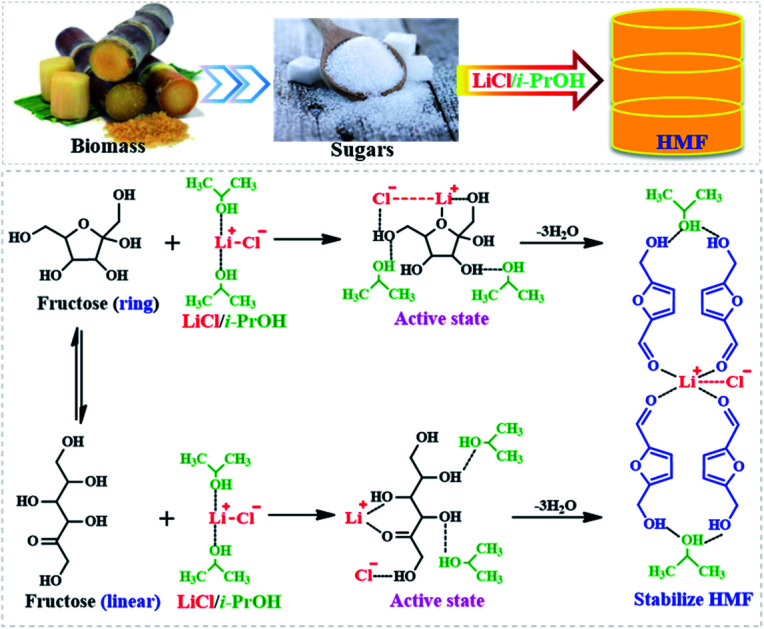

## Introduction

5-Hydroxymethylfurfural (HMF), a well-known six-carbon molecule, is identified as one of the renewable and versatile intermediates for the production of a variety of chemicals and alternative fuels, such as 5-ethoxymethylfurfural, 2,5-furandicarboxylic acid, 2,5-dimethylfuran, and 2,5-bis(alkoxymethyl)furans, most of which are derived from fossil feedstocks traditionally, due to its three functional moieties of hydroxymethyl (–CH_2_OH), formyl (–CHO) and a furan ring.^1^ Undoubtedly, the carbohydrate-derived HMF has been regarded as one of the bridges between conventional fossil resources and renewable carbons.^2,3^ In recent years, catalytic conversion of renewable biomass into HMF has attracted much attention, and developing sustainable and efficient methods and chemical processing is still demanded with the rapidly diminishing supply of fossil feedstock.^4,5^

In general, the six-carbon sugars, such as glucose, fructose, sucrose, and starch, are commonly used as the feedstocks for HMF production. It is found that fructose-based carbohydrates (fructose, sucrose, inulin, *etc.*) are the prevalent feedstocks due to their much easier dehydration process in the presence of acid catalysts.^6,7^ So far, diverse catalysts involving strong mineral acids (HCl, H_2_SO_4_, H_3_PO_4_, *etc.*), strong metal-based Lewis acids (Cr^3+^, Sn^4+^, Fe^3+^, *etc.*), and solid acid catalysts (acidic zeolites, cation-exchanged resins, and others) have been developed for the conversion of fructose-based carbohydrates into HMF.^6,8^ Although these acid catalysts have excellent catalytic performance for carbohydrates conversion, they still suffer from one or more persisting drawbacks such as equipment corrosion (mineral acids), environmental toxicity (Lewis acids), difficulty in product separation and catalyst recycling (mineral acids and Lewis acids), tedious workup procedures (solid acids), and so on.^6,8–10^ Therefore, it is pressing to explore new safer, milder, and less environmentally impactful catalysts that are in line with the concept of sustainable chemistry to catalyze carbohydrates to produce HMF.

However, the search for efficient catalysts frequently leads to new homogeneous and heterogeneous catalysts of increasing complexity, sometimes overlooking common, commercially available materials that can be used in catalysis directly will be more practical. For example, lithium chloride (LiCl) is weak Lewis acid with lower environmental risk, and commonly used as an additive to privileged cellulose solvents, *i.e.*, LiCl/*N*,*N*-dimethylacetamide (LiCl/DMAc).^11^ The Li^+^ and Cl^−^ played the key role for cellulose dissolution in LiCl containing solvent systems, and could keep strong interaction with the hydroxy groups (–OH) attached in cellulose chains.^12–14^ Recently, Binder *et al.*^15,16^ found that the LiCl was an additive for promoting carbohydrates dehydration reactions in the presence of toxic CrCl_2_ or CrCl_3_ catalyst; and with the aid of LiCl, the yield of HMF elevated significantly from fructose, glucose, and cellulose substrates in DMAc solvents; Chen *et al.*^17^ studied the promotion of LiCl on CrCl_2_, SnCl_4_ or SnCl_2_ catalyzed dehydration of glucose into HMF in the caprolactam solvent, achieving acceptable HMF yield of 55–67% with the suitable amount of LiCl additive. Moreover, LiCl could also act as a catalyst in catalytic ring-opening polymerization of lactide in the presence of hydroxyl-containing compounds,^18^ catalytic Friedel–Crafts reaction of electron-rich aromatic compounds with ethyl glyoxylate,^19^ and catalytic coupling reactions of propylene oxide and CO_2_ to produce propylene carbonate (PC) with the help of imidazolium halide.^20^ Most recently, the LiCl has been used as a phase-transfer catalysts to synthesize the thin Co_2_P nanosheets for oxygen evolution reaction,^21^ and has also accelerated the cross-coupling of aryl chlorides with aryl triflates in the presence of Ni/Pd-based multimetallic system.^22^ The outstanding unexpected performance of LiCl in catalysis makes it an alternative additive or promoter for the production of HMF from fructose-based carbohydrates.

As known that HMF is formed from the acid-catalyzed dehydration of hexoses by elimination of three water molecules. Hence, the existence of water not only depressed the dehydration of hexoses into HMF, but also accelerated the rehydration of HMF into levulinic acid (LA) and formic acid (FA), resulting in the lower HMF yield and selectivity.^1,23^ In order to shield the reactive HMF from the aqueous acidic environment, thereby preventing side reactions, the dehydration reactions are usually carried out in non-aqueous solvents, such as dimethyl sulfoxide (DMSO), dimethylformamide (DMF), ionic liquids, or mixtures. Since then, high yields of HMF have been reported in ionic liquids and high-boiling-point organic solvents.^6,23^ However, the high cost, toxicity of the solvents, and the difficulty in recycling the above reaction media due to the problematic isolation of HMF present major challenges for large-scale biorefinery applications.^6,24^ In this context, the low boiling point non-aqueous solvents for efficient conversion of sugars into HMF should be developed. Fortunately, the renewable isopropanol (i-PrOH) was found to be effective to mediate the dehydration reaction efficiently to produce HMF.^25,26^ It has low boiling point and low environmental toxicity, and thus enables a simple production and isolation of HMF, and offers a new opportunity for a large-scale economically viable process.^24,27^ Hence, the i-PrOH will be a promising environment-friendly solvent for HMF production with the LiCl additive. In addition, it is found that the performance of LiCl for catalysis will be improved by the combination of alcohols.^18,19^ It is hypothesized reasonably that the LiCl-promoted and i-PrOH-aided system will be more efficient for conversion of fructose-based carbohydrates into HMF.

Herein, in consideration of all above issues and in connection with our research program centering on the conversion of sugars into HMF,^28–31^ a LiCl-promoted and i-PrOH-aided system for the dehydration of fructose-based carbohydrates into HMF has been constructed consequently. The promotion effect of LiCl, the effect of reaction conditions, the extent of the substrate types are evaluated systematically.

## Experimental

### Materials

d-Fructose (99%), sucrose (99%), inulin (97%), glucose (99%), maltose (99%), starch (99%), cellulose (99%), HMF (99%), and CH_3_OH (high-performance liquid chromatography, HPLC) were supplied by the J&K Chemical Company (Beijing, China); the analytical graded LiF (99%), LiCl (99%), LiBr (99%), LiI (99%), NaCl (99%), KCl (99%), MgCl_2_ (99%), CaCl_2_ (99%), NH_4_Cl (99%), and i-PrOH (99%) were purchased from Sinopharm Chemical Reagent Co., Ltd. (Shanghai, China). All reagents are used as received without further pretreatment.

### Typical procedure for the dehydration of fructose

In a typical experiment, 0.9 g of fructose (10 mmol), 0.42 g of LiCl (10 mmol) and 10 mL of i-PrOH were charged into a 25 mL polytetrafluoroethylene (PTFE) lined hydrothermal autoclave reactor, and then the reactor was flushed with nitrogen (N_2_) stream for 1 min to displace the air. Finally, the reactor was capped hermetically and immersed into an oil bath preheated to the designed temperature (90–130 °C) for reaction with magnetic stirring (500 rpm). When the designed reaction time (0.5–3.0 h) was over, the reactor was cooled down to room temperature in cool water bath.

### Typical analysis procedure

The amount of unreacted fructose and the yielded HMF were determined by using HPLC. After reaction, the resulting mixture was diluted to 100 mL for qualitative and quantitative analysis on an Agilent 1200 HPLC system equipped with a HPX-87H column (300 × 7.8 mm, 5 μm, 5 mM H_2_SO_4_ solution as the mobile phase at 0.6 mL min^−1^) and RID detector for unreacted substrates, and a C18 reversed phase column [250 × 4.6 mm, 5 μm, 1 : 4 (v/v) CH_3_OH/H_2_O as the mobile phase at 0.6 mL min^−1^] and a UV detector (284 nm) for HMF. The unreacted fructose and HMF amounts were determined through the external standard method using commercially available standard substrates. Conversion of fructose (Conv., mol%), yield of HMF (*Y*_HMF_, mol%), and selectivity of HMF (*S*_HMF_, mol%) were calculated according to eqn (1)–(3). The same process was repeated 3 times to minimize error.1
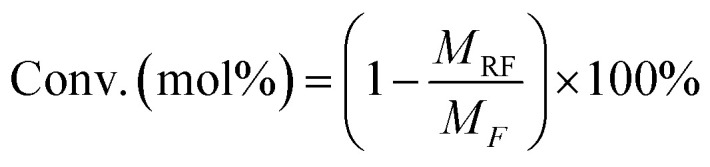
2
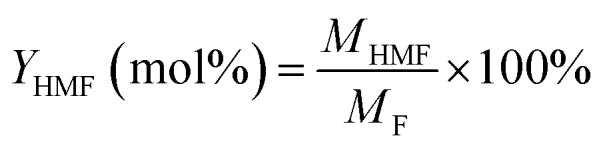
3
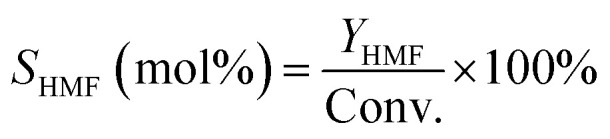
where *M*_F_, *M*_RF_, and *M*_HMF_ are the amounts (mol) of the original fructose, residual fructose, and HMF, respectively.

### Typical procedures for product separation and recycling of LiCl/i-PrOH

After reaction, the i-PrOH was recovered firstly through rotary evaporation due to its low boiling point (82.45 °C, 1 atm), resulting in a mixture of LiCl and HMF as viscous brown liquid. The resulting mixture was extracted with 3 × 10 mL of ethyl acetate (EtOAc) for isolation of HMF. The EtOAc phase containing HMF was collected and evaporated to obtain the crude HMF product as brown oil. The residual LiCl and recovered i-PrOH (dried by Mg_2_SO_4_) could be reused into the next run without further purification (ESI, Scheme S1†).

The structure of HMF was confirmed by proton nuclear magnetic resonance (^1^H NMR) on a Bruker DRX 400 spectrometer using CDCl_3_ as the solvent (ESI, Fig. S1†); and the purity of HMF was around 95% according to the HPLC quantitative analysis (ESI, Fig. S2†). The detailed procedures for the purity analysis of isolated HMF was given in ESI.†

## Results and discussion

### Initial salt additive and solvent testing

It has been reported that the chloride salts (chloride-containing catalysts or additives) have superior performance in synthesis of HMF from carbohydrates. Hence, the promotion of various low or no toxic chloride additives, including LiCl, NaCl, KCl, MgCl_2_, and CaCl_2_, in dehydration of fructose into HMF was studied initially (Table 1).

**Table 1:** Conversion of fructose into HMF with various additives^a^

Entry	Additive	Conversion (%)	HMF	
			Yield (%)	Selectivity (%)
1	LiCl	100	79.1 ± 2.3	79.1 ± 2.3
2	NaCl	82.4 ± 2.2	22.4 ± 1.9	27.2 ± 1.5
3	KCl	99.0 ± 0.6	41.2 ± 1.1	41.6 ± 0.9
4	MgCl_2_	100	0.9 ± 0.1	0.9 ± 0.1
5	CaCl_2_	97.6 ± 2.2	18.2 ± 3.4	18.6 ± 3.0
6	LiF	29.7 ± 2.9	Trace	—
7	LiBr	96.3 ± 1.5	53.1 ± 1.6	55.1 ± 0.7
8	LiI	90.2 ± 2.5	26.7 ± 1.8	29.6 ± 1.2
9	Li_2_SO_4_	40.5 ± 4.3	ND	—
10	NH_4_Cl	100	32.1 ± 2.3	32.1 ± 2.3
11	—	96.3 ± 3.9	6.2 ± 0.9	6.4 ± 1.1

a Conditions: 1.8 g of fructose (10 mmol), 10 mmol of additive, 10 mL of i-PrOH, 130 °C for 2 h.

From Table 1, it could be seen that the above chlorides could promote the conversion of fructose with high conversion degree (>80%); however, only LiCl could afford the good HMF yield of up to 79.1% (entries 1–5). These results showed that the Cl^−^ anions could promote the fructose conversion, and only the Li^+^ could induce the dehydration of fructose into HMF through stabilizing the HMF with association between the Li^+^ cations and the carbonyl groups (–CHO) of HMF.^32^ The contribution of Cl^−^ has also been identified, when the anion of LiCl was replaced by F^−^, Br^−^, I^−^ and SO_4_^2−^, the conversion efficiency was all lower than the LiCl-promoted system (entries 6–9). These results showed that the co-operation of Li^+^ and Cl^−^ was crucial for the dehydration of fructose into HMF in i-PrOH. These attributes Bode well for the use of LiCl as an alternative additive for HMF production. Furthermore, when the reported ammonium salt (NH_4_Cl) with weak Brønsted acidity was used as the additive,^33^ the fructose was converted completely, affording low HMF production efficiency (entry 10), which might be caused by the occurrence of many side-reactions (Maillard reactions) in the presence of sugars, HMF, and NH_3_ from the decomposition of NH_4_Cl under the high reaction temperature.^34,35^ Hence, the LiCl could act as an effective additive to promote the conversion of fructose into HMF in the subsequent experiments. It should be noted that the HMF selectivity was less than 100%. The result could be attributed to the complexity of the reaction and high reactivity of HMF, several side reactions may occur, the most notable of which are the acid-catalyzed HMF rehydration to levulinic and formic acids, HMF self-condensation reactions, and HMF–fructose cross-polymerization, forming soluble and insoluble polymers named humins confirmed by the brown mixture after reaction.^29–31,36^

**Fig. 1:**
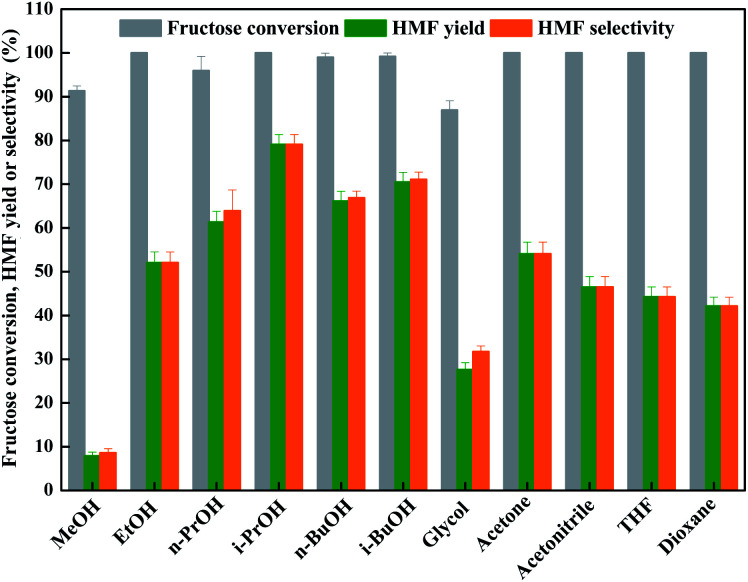
LiCl-promoted dehydration of fructose into HMF in various solvents (reaction condition: 1.8 g of fructose (10 mmol), 0.42 g of LiCl (10 mmol), 10 mL of solvent, 130 °C for 2 h).

As known that the solvent also affected the dehydration process of fructose conspicuously through coordinating or activating intermediates.^7,37–40^ Hence, the effect of organic solvent with different structure on fructose dehydration with an aid of LiCl was investigated inevitably. Fig. 1 showed that the organic solvents did facilitate the dehydration of fructose with high degree (≥90%) in all cases; however, only bulky alcohols, such as *n*-PrOH, i-PrOH, *n*-BuOH, and i-BuOH, could facilitate the formation of HMF from fructose dehydration process, these results are in line with the reported result by Zhang *et al.*,^25^ demonstrating that the dehydration reaction of fructose in bulky alcohols has a better selectivity to HMF.^41^ Hence, the i-PrOH with low boiling point (82.45 °C, 1 atm) and toxicity is the promising solvent for dehydration of fructose into HMF with the aid of LiCl.

It is reported that the impurities, such as metal species (Fe^3+^, Cu^2+^, *etc.*), in the catalytic system will improve the dehydration of fructose,^42^ hence to confirm the potential influence of impurities in the LiCl/i-PrOH system, the inductively coupled plasma optical emission spectrometer (ICP-OES, Optima 8300, PerkinElmer) was used to detect the existence of other metal species, and no metal impurities could be detected in the reaction system. Hence, we can believe that LiCl and i-PrOH are active components for fructose dehydration to produce HMF.

### Effect of process parameters

**Fig. 2:**
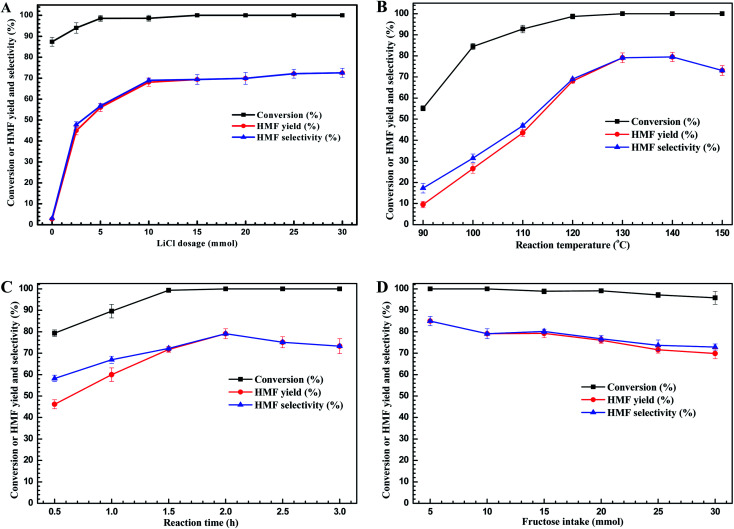
The influence of reaction conditions ((A) 10 mmol of fructose, 10 mL of i-PrOH, 120 °C for 2 h; (B) 10 mmol of fructose, 10 mmol of LiCl, 10 mL of i-PrOH, 2 h; (C) 10 mmol of fructose, 10 mmol of LiCl, 10 mL of IPA, 130 °C; (D) 10 mmol of LiCl, 10 mL of i-PrOH, 130 °C for 2 h).

With appropriate additive (LiCl) and solvent (i-PrOH) in hand, and to find the best experimental conditions to carry out this dehydration reactions, the process parameters such as LiCl loading, temperature, reaction time, and fructose intake were further considered, and the results are present as Fig. 2.

First, we investigated the effect of LiCl loading on HMF yields (Fig. 2A). Without adding LiCl, more than 85% of fructose could be converted into unknown species accompanying with about 2.7% of HMF yielded. This result showed that i-PrOH facilitated the fructose conversion, but not to form HMF. The unknown species have been found to be the intermediates, which could be further converted into HMF with the addition of catalyst according to the controlled experiments. When the LiCl was added into the reaction system, the HMF yield was improved significantly and the conversion degree of fructose could reach 100% at the appropriate LiCl loading (≥5 mmol). For examples, when 2.5 mmol of LiCl was added, the conversion degree of fructose was elevated to 94.0%, however, the HMF yield was improved to remarkable yield of 45.0%, implying the LiCl did promote the formation of HMF from dehydration of fructose. Further increasing the LiCl dosage to 10 mmol, the HMF yield could reach the acceptable value of 68.1%. Moreover, increasing the dosage of LiCl above 10 mmol provided no improvement of HMF yield with complete conversion of fructose. These results above showed that the 10 mmol of LiCl was the sufficient amount for promoting HMF formation through fructose dehydration, and 10 mmol of LiCl was set to the suitable dosage for the subsequent experiments.

Next, we sought to optimize the reaction temperature and duration. Fig. 2B showed that the high reaction temperature facilitated not only the fructose dehydration but also the formation of HMF, when the reaction temperature was elevated to 130 °C, the peak value of HMF yield of 79.1% was achieved. However, further elevating the reaction temperature would cause the lower yield of HMF because of HMF decomposition at elevated temperatures (>130 °C).^29^Fig. 2C showed that at the reaction temperature of 130 °C, the reaction time also played the key role for HMF production. As the reaction time prolonged to 1.5 h, the fructose would be converted completely. Meanwhile, the HMF yield presented an increasing trend as the reaction time prolonged, and reached the peak value of 79.1% at 2.0 h. Further prolonging the reaction time, the HMF yield declined, reflecting the weak stability of HMF at higher reaction temperature (>130 °C) due to some side-reactions occurred as reaction time extended.^30,43^ Hence, to obtain the higher HMF yield, the optimized reaction temperature and time would be set to 130 °C for 2 h.

Last, from a practical and economical point of view, the initial fructose intake is also one of the crucial factors for HMF production in industrial process. Looking at the plot pictured in Fig. 2D revealed that fructose afforded good conversion degree in all cases (∼100%). However, the HMF yield declined with increasing of initial fructose concentration, and at the 30 mmol of fructose intake, the lowest value of HMF yield of 69.9% reached frustratedly. These results could be attributed to the fact that increasing the fructose intake led to higher collision probability of fructose and generation of HMF, leading to self- or cross-polymerization and formation of humins,^31,44^ and thus resulting in the low HMF yield at high fructose concentration. Nevertheless, the HMF yield is still acceptable with the moderate fructose intake, achieving 79.7% of HMF yield at a fructose intake of 10 mmol. Hence, the appropriate fructose intake in this work is set as 10 mmol, which is more meaningful in the process economy.

Therefore, on the basis of the analysis of the process parameters, the optimal conditions were found to be 10 mmol of LiCl dosage at 130 °C for 2.0 h with 10 mmol of fructose intake, affording acceptable HMF yield of 79.1%.

### Kinetics analysis and mechanism hypothesis

**Fig. 3:**
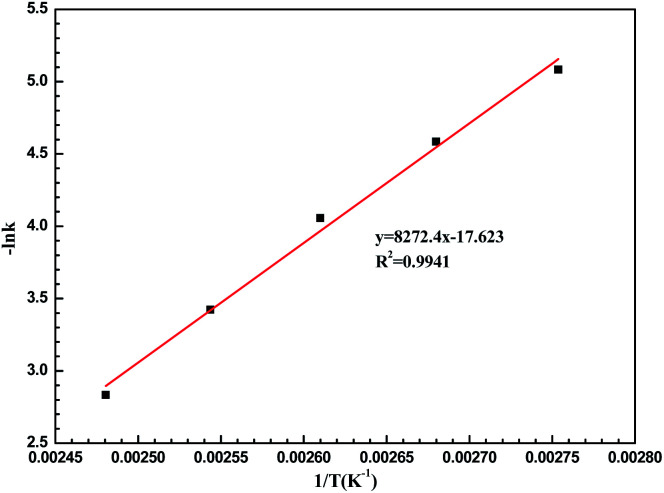
Arrhenius plots for LiCl-promoted fructose dehydration in i-PrOH.

To understand the LiCl-promoted and i-PrOH meditated dehydration of fructose into HMF deeply, the kinetic analysis of this reaction was performed. The activation energy and pre-exponential factor for LiCl-promoted fructose dehydration to HMF could be obtained through Arrhenius plot generated from first-order kinetic constants (Fig. 3, S3 and S4†), and be summarized in Table S1.†

**Scheme 1:**
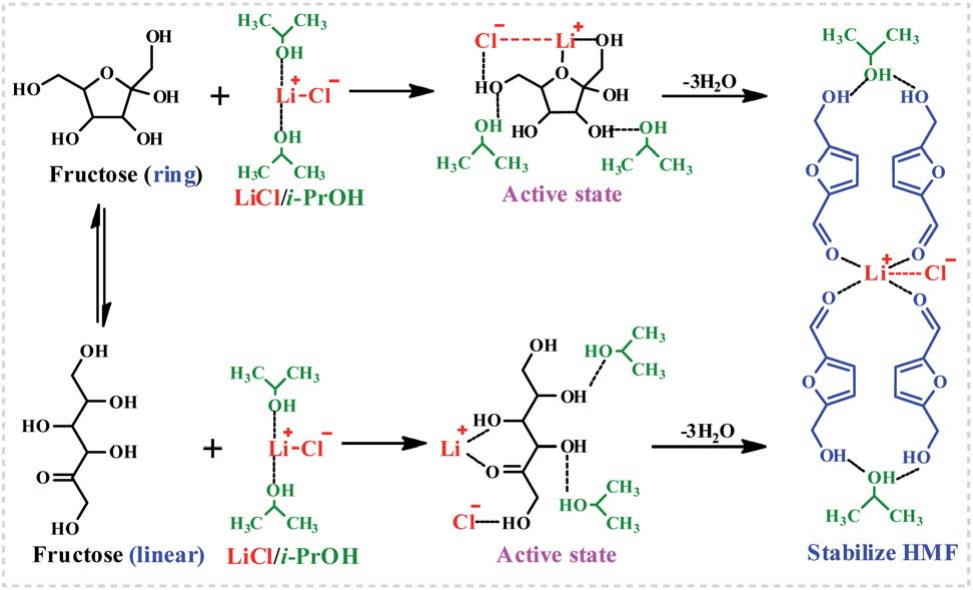
The proposed dehydration mechanism of fructose promoted by LiCl in i-PrOH medium.

It could be found that the activation energy for fructose dehydration in LiCl-promoted and i-PrOH aided system was only 68.68 kJ mol^−1^ with a pre-exponential factor value of 1.2 × 10^4^ min^−1^, this result revealed that the dehydration reaction of fructose occurred easily in the presence of LiCl in i-PrOH (kinetic control), and also implying that the LiCl-promoted and i-PrOH meditated system is suitable for production of HMF from fructose. Furthermore, based on the results above (Table 1, Fig. 1–3, Table S1 and Fig. S3, S4†), the mechanism of this dehydration was also proposed, and illustrated as Scheme 1.

In a word, the efficient production of HMF from fructose in LiCl/i-PrOH system is achieved through the LiCl-promoted and i-PrOH-aided dehydration of fructose into HMF, which is further stabilized by LiCl and i-PrOH through the formation of hydrogen bonds and coordination bonds,^13,32,45–49^ affording satisfactory yield of HMF.

### Substrate scope

To study the generality of LiCl-promoted and i-PrOH aided system for production of HMF, a series of substrates, such as fructose in large scale, glucose, sucrose, maltose, inulin, starch and cellulose, were loaded into this reaction system. It was found that this reaction system was suitable for HMF production in large scale, because when the reaction system was scaled up by 10 time, the acceptable HMF yield of 73.6% could still be achieved (Table 2, entry 2), reflecting the potential practicability of this system for the production of HMF in industrial process.

**Table 2:** Substrate range of this LiCl-promoted system^a^

Entry	Substrate	Conversion (%)	HMF	
			Yield (%)	Selectivity (%)
1	Fructose	100	79.1 ± 2.3	79.1 ± 2.3
2^b^	Fructose	100	73.6 ± 3.1	73.6 ± 3.1
3	Glucose	13.7 ± 1.9	4.2 ± 1.0	30.9 ± 3.2
4	Sucrose	60.5 ± 2.3	30.3 ± 2.2	31.5 ± 1.8
5	Maltose	20.5 ± 2.6	2.8 ± 0.6	13.8 ± 1.1
6	Inulin	—	70.3 ± 1.2	—
7	Starch	0.5 ± 0.1	<0.1	—
8	Cellulose	0	ND	—

a Conditions: sugar units 10 mmol, LiCl 10 mmol, i-PrOH 10 mL, 130 °C for 2 h.

b Sugar units 100 mmol, LiCl 100 mmol, i-PrOH 100 mL, 130 °C for 2 h.

However, this system was only suitable for fructose-based carbohydrates, and when the glucose, maltose, starch, and cellulose were used as the feedstocks, the yield of HMF could be neglected, these results can be attributed to the weak ability of LiCl for catalytic isomerization of glucose into fructose (Table 2, entries 3, 5, 7 and 8), which is the key step for formation of HMF from glucose.^50^ This specialty was confirmed by the sucrose as the raw material due to 30.3% of HMF derived from fructose moiety of sucrose (Table 2, entry 4). Hence, this system showed the positive effect for synthesis of HMF from fructose-based carbohydrates, even though the famous biopolymer of fructose, inulin, used as the feedstock, the excellent yield of HMF (70.3%) could still be achieved (Table 2, entry 6). Therefore, the LiCl-promoted and i-PrOH aided system is the potential candidate choice for production of HMF from fructose-based carbohydrates.

### Recyclability of LiCl and solvent

**Fig. 4:**
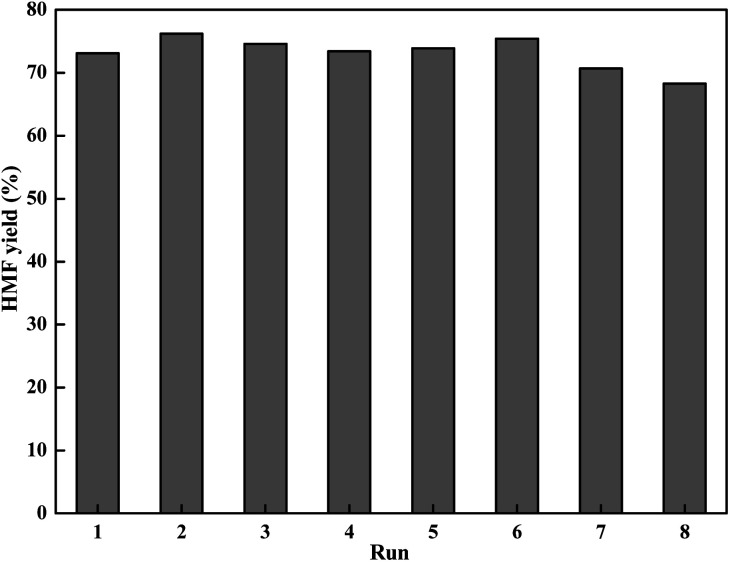
Recycle experiments (reaction condition: 1.8 g of fructose (10 mmol), 0.42 g of LiCl (10 mmol), 10 mL of solvent, 130 °C for 2 h).

Reusability is a critical parameter to evaluate the catalyst property, especially in viewpoint of practical application. With the above information in mind, we investigated the possibility of reusing the LiCl/i-PrOH system for conversion of fructose into HMF (Fig. 4).

As shown in Fig. 4, using recycled solvent and LiCl directly for the dehydration reaction, the yield of HMF is still retained at 68.3% in the 8^th^ cycle, indicating a quite low activity lost in each cycle. It is interesting to see that the yield of HMF in some recycles was even higher than the first one, which can be attributed to the retention of HMF and residual unreacted fructose from the previous cycle.^47,51,52^ This simple product isolation and solvent recycling process makes the reaction system particularly suitable for industrial operation.

## Conclusions

In conclusion, the LiCl-promoted dehydration of fructose-based carbohydrates into HMF has been developed in i-PrOH aided system (LiCl/i-PrOH). Under the optimum conditions, *viz.*, 10 mmol of LiCl dosage and 10 mmol fructose intake at 130 °C for 2.0 h, 79.1% of HMF can be afforded with full conversion of fructose. The LiCl/i-PrOH system could not only promote the dehydration of fructose, but also stabilize the as-formed HMF, giving low activation energy of fructose dehydration (68.68 kJ mol^−1^) with a pre-exponential factor value of 1.2 × 10^4^ min^−1^. The 73.6% of HMF yield could be obtained from the scaled-up LiCl/i-PrOH system (10 time), accompanying with 30.3% and 70.3% of HMF yield from sucrose and inulin feedstocks, respectively. Moreover, this catalytic system could be reused 8 times without significant loss of activity. From the perspective of green and sustainable chemistry, this work demonstrates green benefits not only constructing sustainable catalytic system, but also demonstrating green production of HMF from biomass. The readily available and low-toxic LiCl, the sustainable i-PrOH solvent, the renewable starting materials, and the mild reaction condition make the LiCl/i-PrOH system promising and sustainable for industrial production of HMF in future.

## Conflicts of interest

The authors declare no competing financial interest.

## Supplementary Material

RA-011-D0RA08737H-s001
