# Supplementary material for: LiCl-promoted-dehydration of fructose-based carbohydrates into 5-hydroxymethylfurfural in isopropanol
Source: RSC Adv. 2021 Jan 5;11(3):1404–10. doi: 10.1039/d0ra08737h (PMC8693523; doi:10.1039/d0ra08737h)
Supplement: RA-011-D0RA08737H-s001 [file RA-011-D0RA08737H-s001.pdf]

Electronic Supplementary Material (ESI) for RSC Advances.
This journal is © The Royal Society of Chemistry 2020

Electronic Supplementary Information

LiCl-promoted-dehydration of fructose-based carbohydrates into 5-hydroxymethylfurfural in isopropanol

Hao Ma,*[a] Zhenzhen Li,[b] Yanhui Qiao,[a] and Junjiang Teng[a]

[a] College of Chemistry, Guangdong University of Petrochemical Technology, Maoming 525000, P. R. China

[b] School of Chemistry and Chemical Engineering, South China University of Technology, Guangzhou 510640, P. R. China

Corresponding Author

*E-mail: thma@gdupt.edu.cn (H. Ma)

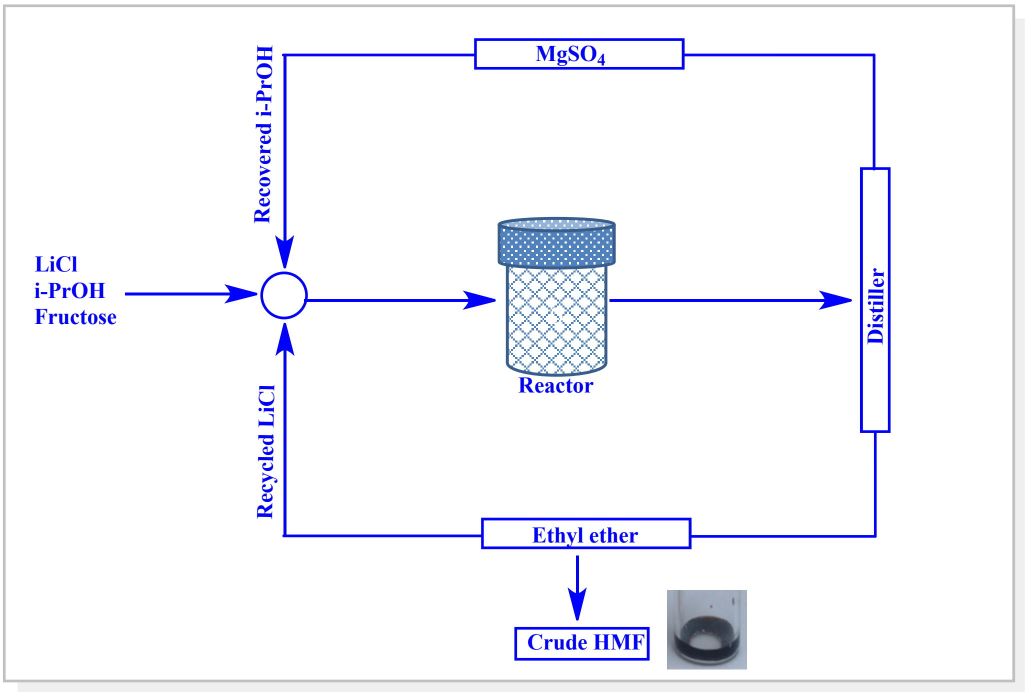

Scheme S1. The procedures for recycling of LiCl/*i*-PrOH system

Typical analysis procedure for the purity of isolated HMF

A certain amount of the isolated HMF (M_1, g) was diluted to 100 mL with deionized water for quantitative analysis on an Agilent 1200 HPLC system equipped with a C18 reversed phase column [250 × 4.6 mm, 5 μm, 1:4 (v/v) CH_3OH/H_2O as the mobile phase at 0.6 mL/min] and a UV detector (284 nm). The HMF amount was determined through the external standard method using commercially available HMF standard. Purity of the isolated HMF (P_{HMF}, mol%) was calculated according to equation S1. The same process was repeated 3 times to minimize error.

$$P_{HMF}(mol\%) = \frac{m_{HMF} \times 126.11}{M_1} \times 100\% \qquad (S1)$$

where M_1 is the mass (g) of isolated HMF used for purity analysis; m_{HMF} is the amount (mol) of HMF existed in the diluted solution (100 mL) from HPLC analysis; 126.11 is the molecular weight (g/mol) of HMF.

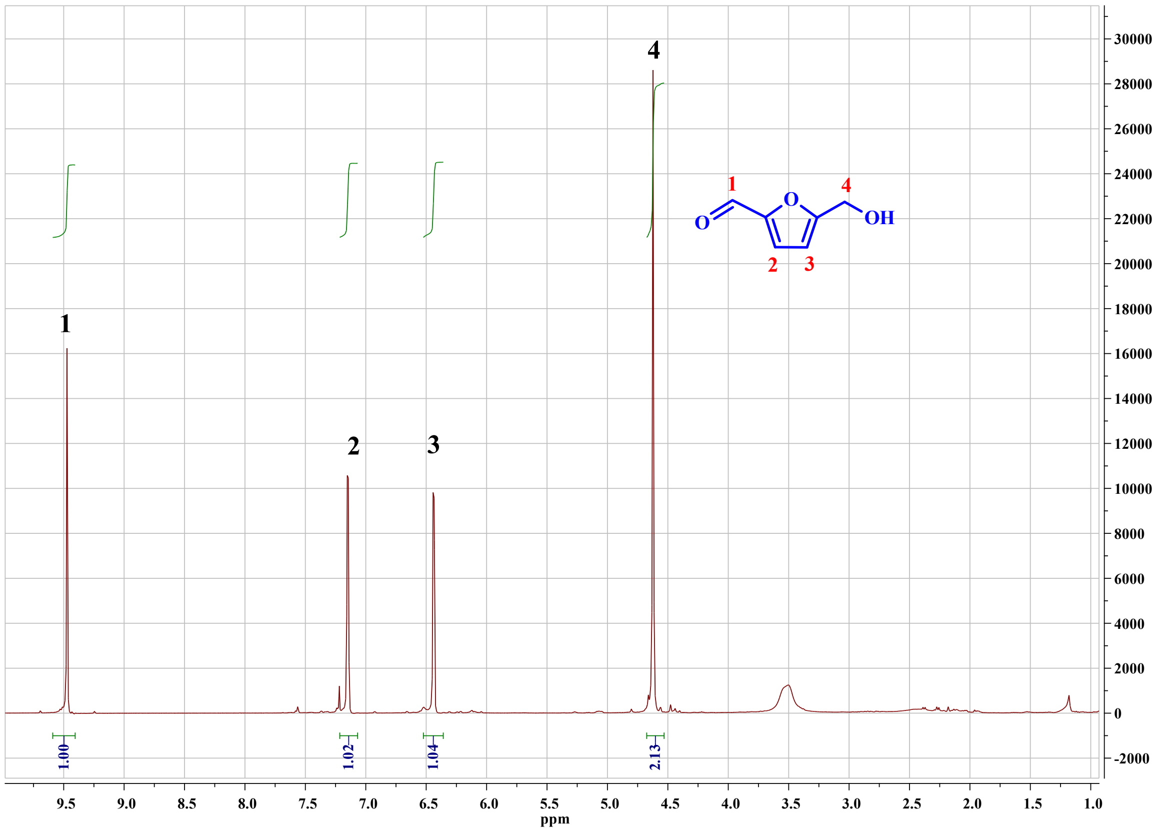

Fig. S1 NMR spectrum of the isolated HMF (400 MHz, $CDCl_3$ as the solvent)

[δ 9.48 (s, 1H, CHO), 7.16 (d, 1H, CH), 6.45 (d, 1H, CH), 4.63 (s, 2H, CH_2)]

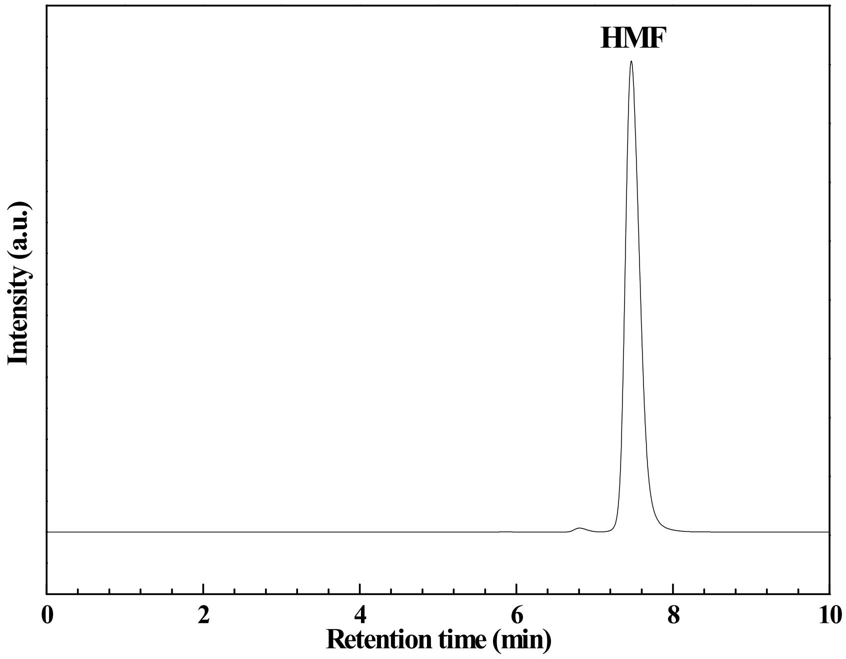

Fig. S2 HPLC profile of the isolated HMF

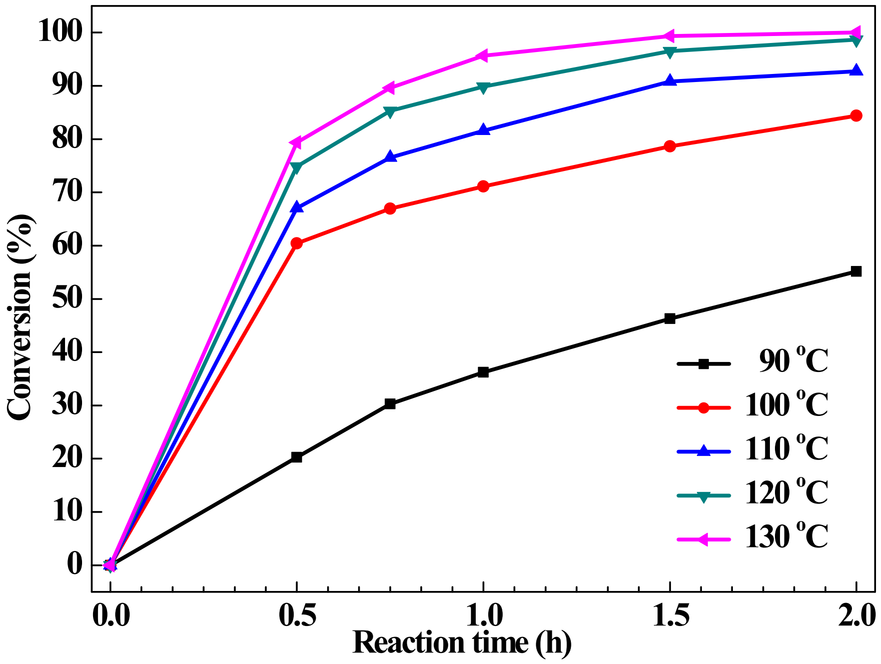

Fig. S3 Effect of reaction temperature and time on the conversion of fructose (1.8 g of fructose (10 mmol), 0.42 g of LiCl (10 mmol), 10 mL of *i*-PrOH).

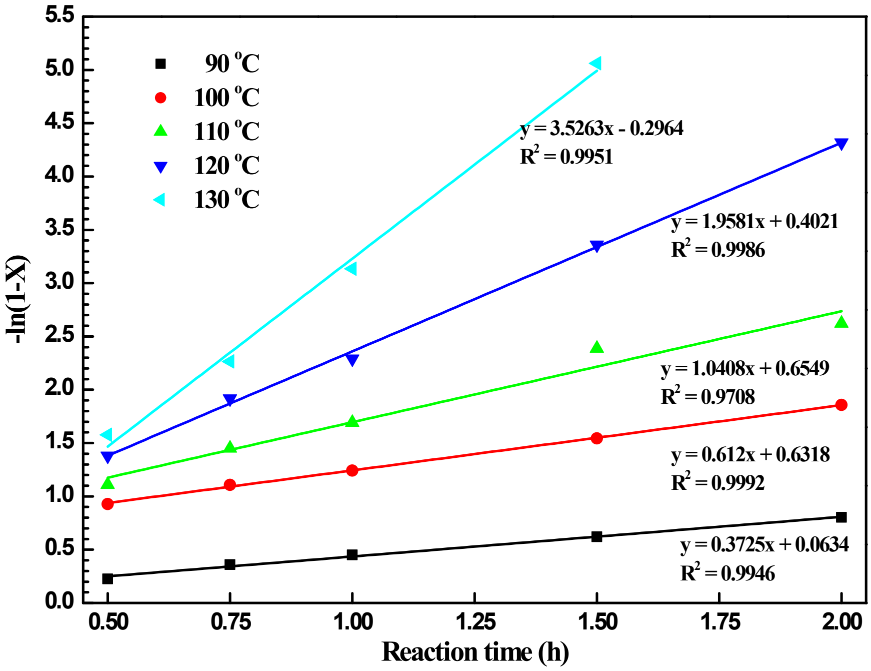

Fig. S4. Reaction rate constant of fructose decomposition at different temperature (1.8 g of fructose (10 mmol), 0.42 g of LiCl (10 mmol), 10 mL of *i*-PrOH).

$$X = (1 - \frac{\text{amount of residual fructose (mol)}}{\text{amount of feed fructose (mol)}})$$

Table S1 Activation energies and pre-exponential factors

Parameter	Values
Reaction order, *n*	1.0
Activation energy, *Ea* (KJ·mol^{-1})	68.68
Pre-exponential factor, *A* (min^{-1})	1.2×10^{4}
Correlation coefficient	0.9941
